# Prosocial emotions predict individual differences in economic decision-making during ultimatum game with dynamic reciprocal contexts

**DOI:** 10.1038/s41598-024-62203-y

**Published:** 2024-05-18

**Authors:** Jaewon Kim, Su Hyun Bong, Dayoung Yoon, Bumseok Jeong

**Affiliations:** Graduate School of Medical Science and Engineering, Korea Advanced Institute of Science and Technology (KAIST), 291, Daehak-Ro, Yuseong-Gu, Daejeon, 34141 South Korea

**Keywords:** Prosocial emotions, Reciprocity, Social decision-making, Personality, Individual differences, Social interaction, Human behaviour, Cooperation

## Abstract

Social decision-making is known to be influenced by predictive emotions or the perceived reciprocity of partners. However, the connection between emotion, decision-making, and contextual reciprocity remains less understood. Moreover, arguments suggest that emotional experiences within a social context can be better conceptualised as prosocial rather than basic emotions, necessitating the inclusion of two social dimensions: focus, the degree of an emotion's relevance to oneself or others, and dominance, the degree to which one feels in control of an emotion. For better representation, these dimensions should be considered alongside the interoceptive dimensions of valence and arousal. In an ultimatum game involving fair, moderate, and unfair offers, this online study measured the emotions of 476 participants using a multidimensional affective rating scale. Using unsupervised classification algorithms, we identified individual differences in decisions and emotional experiences. Certain individuals exhibited consistent levels of acceptance behaviours and emotions, while reciprocal individuals' acceptance behaviours and emotions followed external reward value structures. Furthermore, individuals with distinct emotional responses to partners exhibited unique economic responses to their emotions, with only the reciprocal group exhibiting sensitivity to dominance prediction errors. The study illustrates a context-specific model capable of subtyping populations engaged in social interaction and exhibiting heterogeneous mental states.

## Introduction

Multiple factors influence human decision-making. Neuroeconomic studies have consistently demonstrated that punitive reactions increase as the perceived fairness of a partner decreases^1–3^. This relationship finds support in the reinforcement learning (RL) literature, wherein reward prediction error (PE) has been shown to predict human decision-making^4,5^.

Emotion is another important component of human decision-making. Empirical studies have indicated the influence of predictive emotion on human decision-making^6–9^. Decision affect theory posits that humans anticipate emotions prior to decision-making (predictive emotion). After a decision is made, actual experienced emotions combine the outcome of decisions, counterfactual comparison to unchosen options, and the likelihood of the outcome. Recently, the violation of expectations about emotions was found to impact human decision-making^10^. These findings suggest that predictive emotions modulate the relationship between objective reward value associated with options and actual decision-making.

Social decision-making is frequently investigated using an ultimatum game (UG). In a UG, a proposer makes an offer to a responder who decides to accept or reject this offer. If accepted, both players receive the reward that the proposer splits. If rejected, none of the players receive the reward. In this latter case, the responder engages in what is termed altruistic punishment by exerting pressure on the proposer to behave cooperatively, despite the potential cost this may impose on the responder^11^.

Studies using a UG task have shown that decision-making in a social context is respectively influenced by perceived fairness^1,3^, predictive emotions^10,12^, and emotional feedback from partners^13–15^. However, the comprehensive relationships between social decisions, emotions, reciprocity, and individual differences in those relationships are still unknown.

Simultaneous ratings of affect during UG, referred to as dynamic affective representation mapping (dARM), facilitate tracing momentary emotions during UG social decision-making. It has been suggested that emotional experiences are context-dependent, with individual differences in emotional responses to reward PE and the representation of each emotion criterion^10^.

Emotional experiences paired with social context can be better examined as prosocial emotions^16,17^ than basic emotions^18^. Basic emotions are represented by the two dimensions of valence and arousal^19^. A modified version of James-Lange theory of emotion suggests that these dimensions encode interoceptive information, with hedonic valence information processed by the orbitofrontal cortex and arousal information processed by the anterior insula^20^. However, the adequacy of this two-dimensional (2D) affective representation for prosocial emotions remains uncertain.

In the realm of emotional theory, the Jamesian perspective conceptualizes emotions as integrated bodily state mappings. Concurrently, the somatic marker hypothesis suggests that interoception empowers individuals to differentiate between self and non-self, thereby influencing intuitive decision-making and emotional biases^20,21^. Furthermore, The higher-order theory of emotion posits a convergent viewpoint, asserting that emotions are inextricably intertwined with autobiographical information, encapsulated by the notion of “no self, no emotion”^22^. Similarly, the theory of constructed emotions contends that emotions are socially generated constructs of affective experiences, necessitating an understanding of their source and the elements of control^23^.

Hence, two other affective dimensions have been proposed for classifying prosocial emotions. Tangney et al. leveraged the dimension of focus, which refers to the degree of an emotion’s relevance to oneself or others, to classify nine prosocial emotions^17^. Another dimension proposed was dominance, which is the degree to which an emotion feels in or out of control to a person^24^.

Recognizing that social context can provoke prosocial emotions and that utilizing a two-dimensional affective representation is insufficient, emotions in social context can be better explored using four dimensions: valence, arousal, focus, and dominance. Whether these candidate features are appropriate for representing prosocial emotions and subtyping individuals’ experiences of them should be explored^25^.

This study aims to establish a representation of individual emotional experiences and decision-making during ultimatum game. We hypothesised that individuals could be clustered into subpopulations according to their distinct trajectories of UG social decision-making, as measured by the binary decision of accepting monetary rewards. Furthermore, emotional experiences during UG social decision-making were represented by valence, arousal, focus, and dominance. By clustering multidimensional emotion experiences during UG, we examined the utility of these dimensions in subtyping populations based on the trajectory of emotions. Finally, we examined the differential relationship between emotional experience and UG social decision-making among subtypes. It is suggested that hierarchical account that assumes homogeneity of parameters in subpopulation, can identify individual differences in cognition and behaviours^26^. Utilizing the emotion subpopulation as group label, we illustrate a hierarchical model of emotion and social decision-making that can aid in identifying and predicting individual social decisions influenced by emotions.

## Methods

### Ethics statement

This study was reviewed and approved by the Korea Advanced Institute of Science and Technology (KAIST) Institutional Review Board, Assurance # KH2021-084. In compliance with the guidelines set forth by the KAIST IRB, under protocol # KH2021-084, all participants provided their informed consent. The study adhered to the editorial and publishing policies of Scientific Reports regarding research involving human participants and identifying information, as well as the Declaration of Helsinki.

### Participants

For this exploratory study, we determined the target sample size of 500 participants using a heuristic approach. This exceeded the sample size of the previous study^10^ and reflects a deliberate yet flexible estimation rather than derivation from traditional statistical power analyses. This decision underscores our intent to prioritize broad investigation over precise effect size predictions, aligning with the exploratory objectives of this study. A power analysis was conducted post-experiment, and the detailed information is provided in the supplementary material.

A total of 636 participants were initially recruited through Prolific, an online psychological experiment platform (URL: https://www.prolific.co/). Among them, 89 returned before completing the emotion classification task (ECT), and 51 did not complete all experiments. Further, 19 participants retracted their consent upon completing the task, and one participant did not provide consent. Thus, 476 participants were considered for the subsequent analyses. In the pre-screening phase, 113 individuals consented to the use of their demographic data but opted not to participate in the main study. Therefore, demographic information for 523 participants was de-identified and analysed in compliance with Prolific’s regulations (Table 1). Nationalities were classified into regions according to Standard Country or Area Codes for Statistical Use (M49)^27^.

**Table 1:** Demographic summary of participants.

Number of participants	N = 523
Age (N = 523)	32.0 (10.0)
Gender (N = 521)	
Man	259 (49.7%)
Woman	248 (47.6%)
Non-binary	14 (2.7%)
Education (N = 521)	
Doctoral	11 (2.1%)
Master	122 (23.4%)
Bachelor	192 (36.8%)
College	55 (10.6%)
High school	113 (21.7%)
Etc	28 (5.4%)
Ethnicity (N = 523)	
White	287 (54.9%)
Black	137 (26.2%)
Asian	22 (4.2%)
Other	29 (5.5%)
Mixed	48 (9.2%)
Relationship (N = 514)	
Seeing/engaged	206 (40.1%)
Single	172 (33.5%)
Married/civil partner	120 (23.3%)
Separated/divorced/widowed/etc	16 (3.1%)
Employment (N = 520)	
Full-time	297 (57.1%)
Part-time	85 (16.4%)
Unemployed	73 (14.0%)
Not in paid work	26 (5.0%)
Due to starting a new job	8 (1.5%)
Etc	31 (6.0%)
History of mental health condition or illness (N = 523)	106 (20.3%)
History of long-term health condition or disability (N = 523)	106 (20.3%)
Regions (N = 523)	
Europe	243 (46.5%)
Africa	168 (32.1%)
America	83 (15.9%)
Oceania	22 (4.2%)
Asia	7 (1.3%)

Values are in either mean (SD) or number of participants (%).

### Experimental design

**Figure 1:**
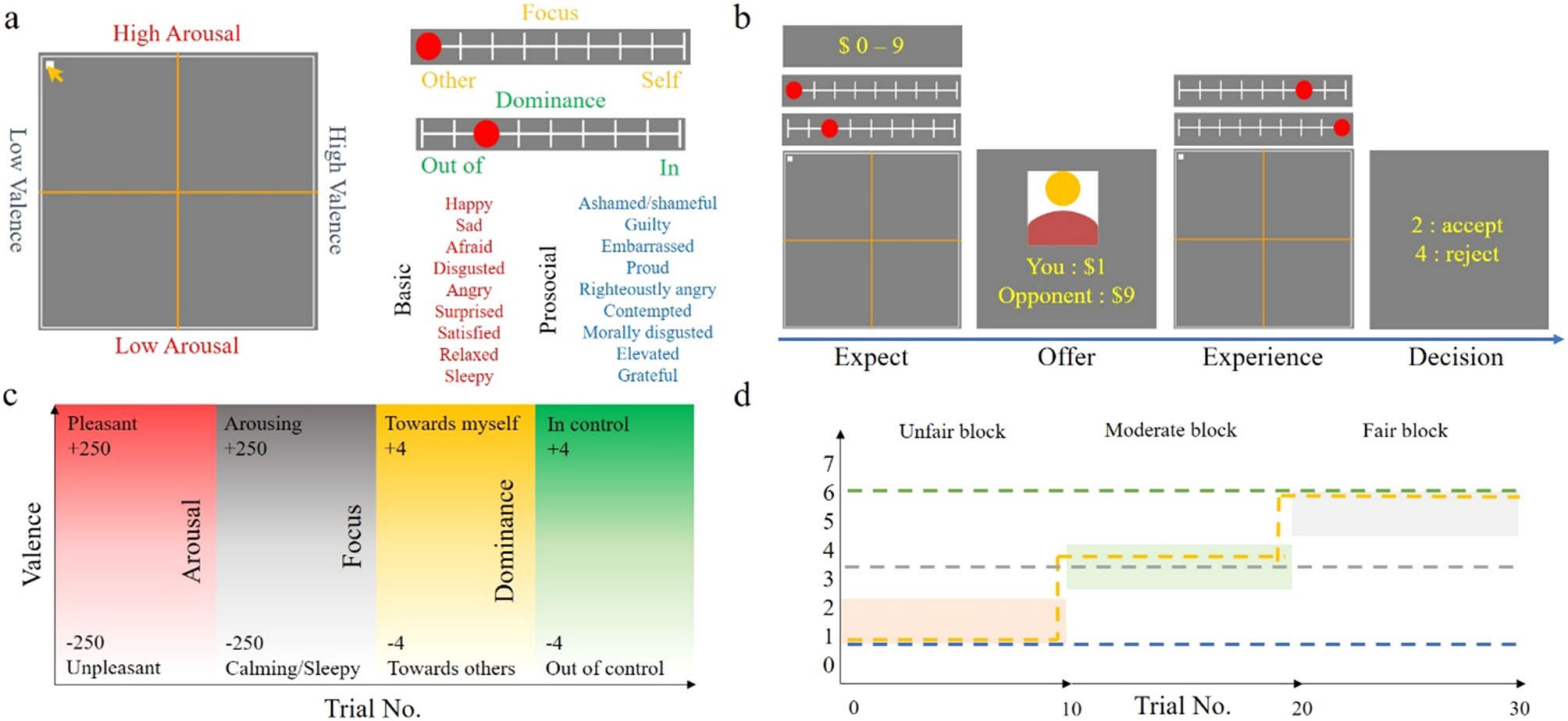
Diagrams of experimental procedures. (**a**) During the ECT, the participants rate basic and prosocial emotions using affective dimensions. (**b**) Within each trial, participants sequentially experience four phases: (1) forming expectations about the proposer’s offer and anticipating their emotional reaction to it, (2) receiving the actual offer from the proposer, (3) rating the emotions they experience upon receiving the offer, and (4) making a decision to accept or reject the proposer’s offer. (**c**) Conceptual diagram of each affective dimension and its gradient. (**d**) Rewards are drawn from pseudorandomised values corresponding to three different fairness levels represented by colored boxes. Examples of potential response trajectories are indicated using dashed lines.

The participants engaged in an in-house computerised cognitive task comprising three stages: ECT, dARM with UG, and clinical rating questionnaires. The task design was based on that of Heffner, Son, and FeldmanHall (2021)^10^, Heffner, FeldmanHall (2022)^12^ and utilised open-source PsychoPy programs that were modified by the authors^28,29^ (Fig. 1). Details of experimental procedures are provided in the Supplementary Text.

### Emotion classification task

At the beginning of the experiment, the participants rated 18 different discrete emotions using valence, arousal, focus, and dominance (Fig. 1A). Nine basic and prosocial emotions were considered to be of interest. Eight basic emotions, except for fear, were selected from the octant of emotion classification plots for enhanced separation^12^. Fear was also included, as five labels overlapped with six basic emotions^18^. The nine prosocial emotions were sourced from Tangney, Stuewig, and Mashek^17^ (Supplementary Table S1).

Because the input format could have influenced the responses, valence and arousal were acquired using a traditional 2D affect grid with 501 × 501 resolution. This grid had horizontal and vertical valence and arousal axes, respectively. Focus and dominance were obtained separately using individual 9-point Likert scales, ranging from − 4 (completely toward others) to + 4 (completely toward myself) for focus, and from − 4 (completely out of control) to + 4 (completely in control) for dominance. The definitions of each affective dimension and emotion label were explained before the experiment.

### Dynamical affective representation mapping with ultimatum game

The participants underwent 30 trials of the one-shot UG as responders. The trials were divided into three blocks of 10 trials, each with different levels of fairness: unfair (mean offer $0.9), moderate (mean offer $2.8), and fair (mean offer $4.9) blocks with a range of offers $0–7. The rewards in each block were pseudorandomised values with a standard deviation of 1.0. Blocks were presented in a random order. The participants were instructed that they would interact with a new person in each trial and that the blocks represented distinct groups. Unique human-like figures were presented for each block. The earned credits were provided to participants in cents ($1.00 = 1 cent). Because clustering algorithms could not handle missing values, a predictive mean matching algorithm was used to impute the missing values in rewards and affective dimensions using R’s mice() package. The choice of predictive mean matching was because of its robustness against misspecification of the imputation model. Analyses were performed after realigning the randomised blocks in an ascending order of fairness.

At the beginning of each trial, the participants were asked to respond with expected rewards (range of 0–9) and the affective dimension they would feel if given the expected amount (Fig. 1B). Subsequently, the offers were given to the participants. After being presented with the offer, the participants rated their current affective dimensions and decided to accept or reject it. The effect of fairness order on the overall acceptance proportion (p_accept_) was tested using ordinary least squares regression.

### Clinical ratings questionnaires

Upon completing dARM with UG, the participants rated their level of depression (Patient Health Questionnaire 9, PHQ-9)^30^, anxiety (Generalised Anxiety Disorder 7, GAD-7)^31^, state anxiety (State-Trait Anxiety Inventory, STAI-X-1)^32^, emotion regulation (Emotion Regulation Questionnaire, ERQ)^33^, and shame and guilt (Personal Feelings Questionnaire 2, PFQ-2)^34^ (Supplementary Table S2). Missing values in clinical ratings were imputed with the average of each participant’s valid responses. The participants’ mean scores of depression, anxiety, and state anxiety corresponded to moderately severe, moderate, and moderate levels, respectively.

### Unsupervised clustering of reward acceptance and experienced emotions

K-means clustering was applied to the entire time series of participants’ expected reward and reward acceptance. Considering the total within the sum of squares values, K = 4 was selected (Supplementary Figs. S1–2).

Simple partitional clustering algorithms were inapplicable because the data of the expected and experienced emotions was multidimensional and changed over time. We classified the emotion time series by applying t-distributed stochastic neighbor embedding (t-SNE) on the bottleneck layer output of the autoencoder using TensorFlow^35,36^. Similar to reward, four clusters were identified (see Supplementary Figs. S3–4). One-way ANOVA and post hoc Tukey HSD tests were applied to compare the expected and experienced emotions between groups (Supplementary Table S3).

### Embedding reward acceptance trajectory onto experienced emotion state space

Uniform manifold approximation and projection (UMAP), a dimensionality reduction algorithm, was applied to the time series of expected and experienced emotions^37^. Subsequently, kernel density estimation plots were separately visualised for each emotion group using Python’s matplotlib and seaborn packages. Consequently, the clustering of subjective emotion experiences based on individual emotion trajectories was observed.

Owing to the similarity in the temporal behaviour of reward acceptance and emotional experience, we tested whether the two variables exhibited a nested structure. To that end, we plotted the reward acceptance trajectory group along the UMAP emotion experience state space. If external reward values can substitute emotion, embedding reward acceptance trajectory along the state space of emotion experience would be identical to the emotion experience embedding results. If emotion experience exhibits independent variance that the reward acceptance trajectory cannot explain, the embedding results would be divergent.

### Generalised linear mixed model to predict social decision-making

To replicate the results of Heffner, Son, and FeldmanHall^10^ and compare the predictive ability of our novel multidimensional dynamic ARM measure with that of a traditional affect grid, a generalised linear mixed model (GLMM) was applied to the participants’ decision for each trial as a dependent variable. PEs for reward, valence, arousal, focus, and dominance were used as predictors, with participants as the grouping variables. This design was adopted from previous work and facilitated the distinguishing between the contributions of reward and emotion PEs during UG social interactions (Eq. 1). The models were nested based on the magnitude of Akaike Information Criteria (AIC) value reduction and compared using AIC for predictive accuracy^38^ (Supplementary Table S4). The significance of model comparisons was tested via likelihood ratio tests. The beta coefficients for each predictor in the winning models were evaluated to test the significance of contributions from each PE term through proportional Z-tests.

1 $${\text{M5a}}:{\text{ Decision }}\sim {\text{ Reward PE }} + {\text{ Valence PE }} + {\text{ Arousal PE }} + {\text{ Focus PE }} + {\text{ Dominance PE }}|{\text{ participants }} \ldots$$

## Results

### K-means clustering algorithm to detect individual differences in social decision-making based on increasing offer fairness

**Figure 2:**
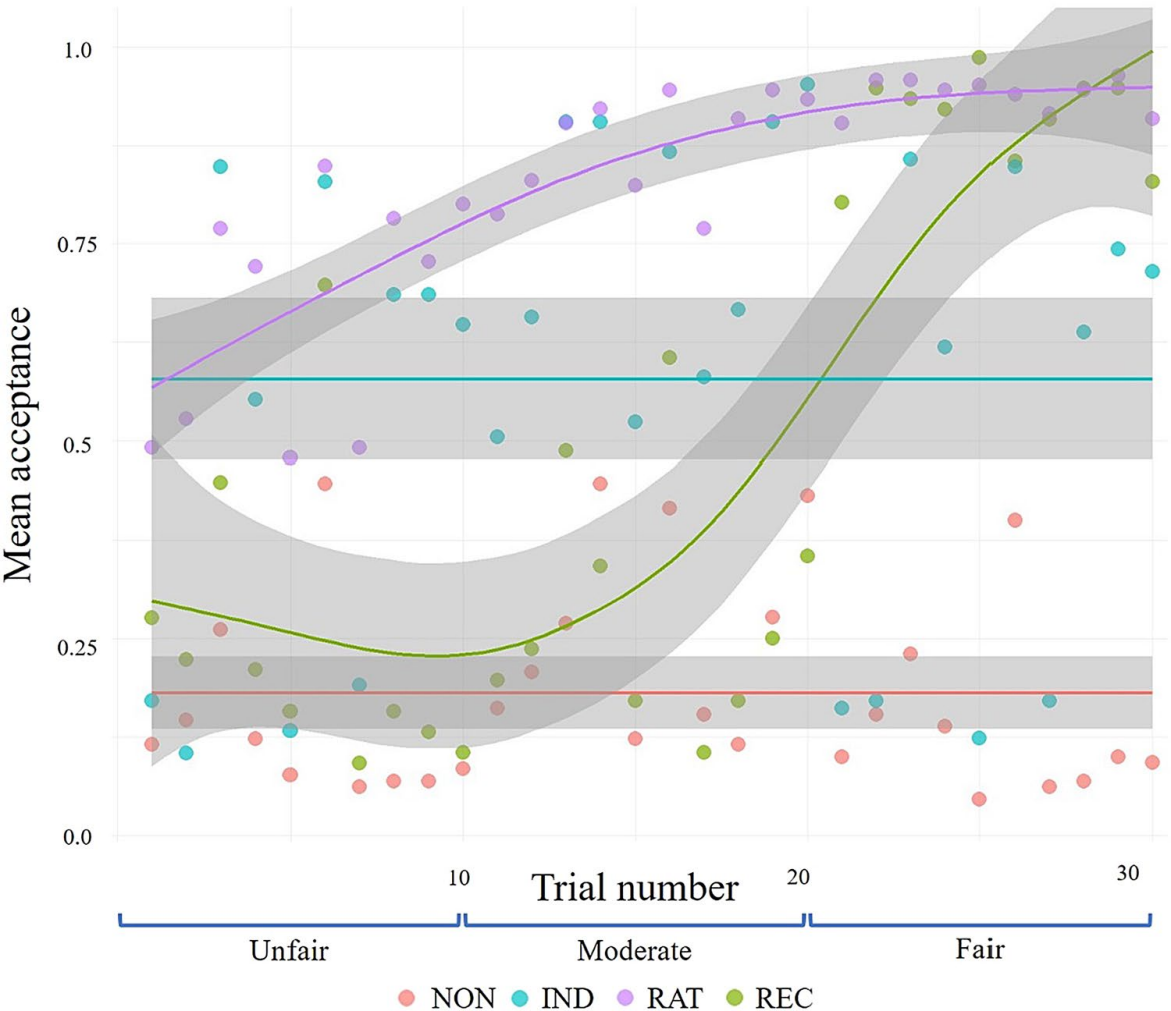
Individual differences in the trajectory of UG social decision-making. Cluster centroids are plotted for each cluster owing to the application of k-means on participants’ acceptance of a given reward. *NON* non-cooperative group, *IND* indifferent group, *REC* reciprocal group, *RAT* rational group.

Ordinary least squares regression revealed no significant influence of fairness order on acceptance proportion (p_accept_). Participant clustering based on their trajectories of reward acceptance resulted in solutions where cluster centroids exhibited three distinct stationary trajectories characterised by consistently low, middle, and high values, along with a unique dynamic trajectory aligning with the actual reward (Fig. 2). We named these clusters as non-cooperative (NON), indifferent (IND), rational (RAT), and reciprocal (REC) groups, respectively.

The mean acceptance ratios for each reward acceptance group were 0.18 (NON, N = 130), 0.58 (IND, N = 105), 0.83 (RAT, N = 165), and 0.48 (REC, N = 76). A one-way ANOVA indicated significant differences in p_accept_ among reward acceptance groups (F = 36.37, *p* < 0.001). A post hoc TukeyHSD test confirmed significant differences between all group pairs except for the IND–REC comparison (Supplementary Table S5). Thus, the clusters exhibited distinct temporal patterns and an estimated decision-making summary.

### Autoencoder neural network classifier identification of central tendency of emotions based on proposer’s offer among individuals

**Figure 3:**
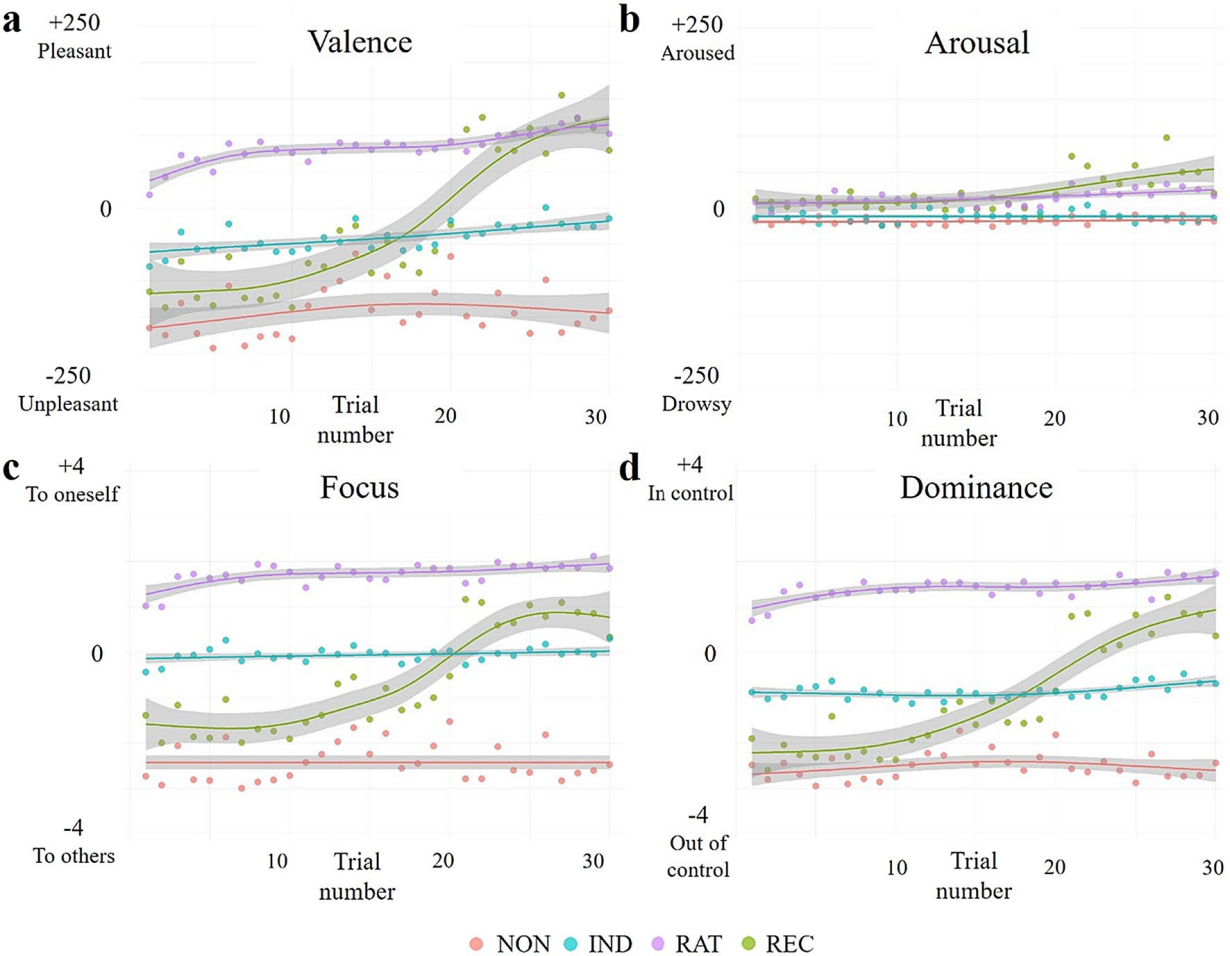
Context-dependent emotion experiences during UG social decision-making. Cluster centroids of emotions are plotted for each cluster using the autoencoder classifier. Each colored line represents one of the four groups. Groups of the same colour across (**a**–**d**) represent the same groups. *NON* non-cooperative group, *IND* indifferent group, *REC* reciprocal group, *RAT* rational group.

Leveraging an autoencoder neural network classifier, we identified individual differences in emotional responses to the proposer’s offer (Fig. 3). Participants’ emotional experiences were classified into four clusters, which exhibited behaviours similar to those of reward acceptance clustering; that is, three groups experienced consistently low, intermediate, and high emotions and one group whose emotion trajectory was chasing the reward structure. Participants experienced a limited range of arousal, unlike the other three affect dimensions.

**Figure 4:**
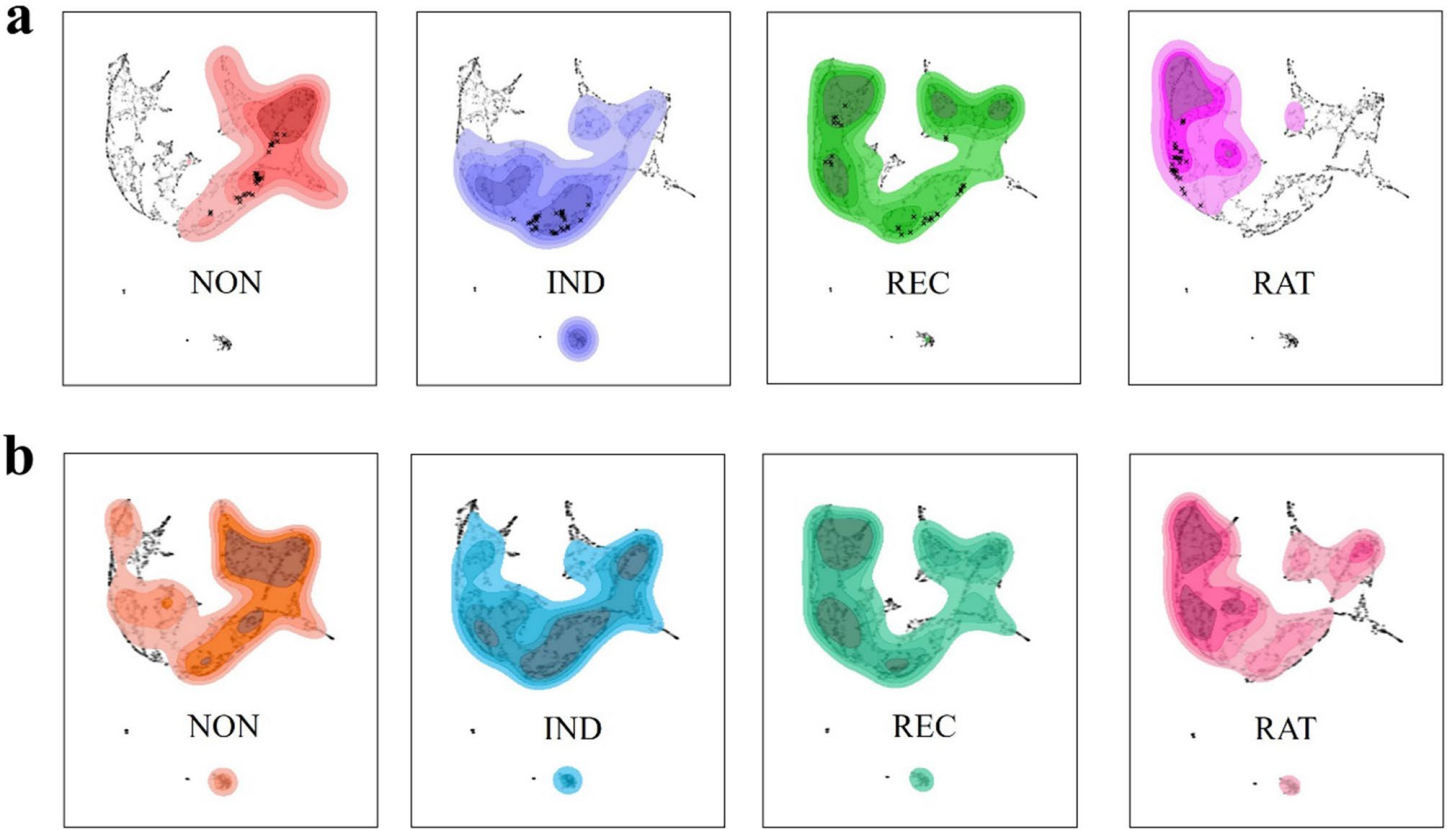
Reward and emotion resemble but are also distinct. The trajectories of experienced emotions were reduced to two components using UMAP. Kernel density estimation was plotted for (**a**) experienced emotion groups and (**b**) reward acceptance groups. Notably, each reward acceptance group occupied a region similar to that of the corresponding emotion group. The same parameters were applied for (**a**) and (**b**), including grid size = 600, levels = 5, thresh = 0.3, and alpha = 0.8. *NON* non-cooperative group, *IND* indifferent group, *REC* reciprocal group, *RAT* rational group.

Embedding the reward acceptance trajectory along the reduced state space of emotion experience indicated alignment with and dispersion from the spatial representation of emotion experience groups. The stationary reward groups (NON, IND, RAT in Fig. 4) occupied regions similar to that of the matched emotion groups, whereas the dynamic groups (REC in Fig. 4) exhibited similar patterns of more dispersed spatial representations that spanned the entire range of the UMAP emotion space.

### Emotion PEs with prosocial dimensions and reward PE predict UG decisions

GLMM results revealed that model M5a, which incorporated all emotion and reward PEs, explained the participants’ responses significantly better than the other models with simpler structures. A likelihood ratio test indicated significant improvement in the explanatory power when the reward, dominance, focus, and arousal PEs were sequentially added to the valence PE (*χ*^2^(4) = 495.83, P < 0.001, *χ*^2^(5) = 350.57, P < 0.001, *χ*^2^(6) = 131.75, P < 0.001, and *χ*^2^(7) = 89.37, P < 0.001).

Estimation of the beta coefficient of each predictor showed that the participants rejected more often when they received a smaller reward and felt less pleased and less in control than expected (Table 2). The valence PE outperformed all other PEs, including the reward PE. The dominance PE indicated a relatively small contribution. Thus, the participants adjusted their decisions most significantly when they experienced greater unexpected (dis)pleasure. Their adjustments were less pronounced in response to reward PE and least pronounced after dominance PE, respectively. PEs along the arousal and focus dimensions did not influence decisions.

**Table 2:** Prediction errors on reward, valence, and dominance as predictors of decisions to accept offers during UG.

Variable	Beta estimate	Proportional Z-test	
		Z	P
Intercept	1.31 (0.12)	10.65	< 0.001
Reward PE	0.60 (0.06)	9.49	< 0.001
Valence PE	0.91 (0.09)	10.12	< 0.001
Arousal PE	− 0.01 (0.06)	− 0.18	0.856
Focus PE	0.11 (0.08)	1.41	0.159
Dominance PE	0.21 (0.07)	2.81	0.005

PE terms are calculated by subtracting the expected value from the experienced values (e.g., Reward PE = experienced reward − expected reward). All variables were scaled but not mean-cantered. The model includes participant-specific random intercepts and slopes for reward and emotion PEs. This analysis comprised 14,280 observations from 476 participants. Bobyqa optimiser was utilised.

The predictors of the model did not show high intra-individual-level correlations (Supplementary Table S6), and their variance inflation factor (VIF) statistics indicated low collinearity with one another (Supplementary Table S7). These results support the reliability of the relationship between the reward PE, emotion PEs, and the participants’ social decisions.

### Individuals differed in their economic responses to predictive emotions.

Individuals experienced different emotional trajectories in response to identical external rewards. Further, participants’ sensitivity to predictive emotions in social decision-making was unique in each affective dimension. Consequently, we investigated whether individuals with different emotional trajectories exhibited unique relationships between predictive emotions and UG social decision-making.

Each emotion experience group was subjected to GLMM analysis. Beta coefficient profiles for each emotion group confirmed individual differences in sensitivity to predictive emotions during UG social decision-making (Table 3). The non-cooperative individuals responded only to valence PE, whereas the rational individuals changed their decisions in response to reward PE. The indifferent group responded either to reward PE or valence PE. Participants in the reciprocal group changed their decisions in response to reward PE, valence PE, and dominance PE. The contributions of the focus and arousal PEs to the social decision were insignificant in any group. The predictive ability of the winning models was generally good, with all area under the curve (AUC) values exceeding 0.9 (Supplementary Fig. S8).

**Table 3:** Summary of GLMM coefficients for each experienced emotion group.

	B0	B1 (RPE)	B2 (VPE)	B3 (APE)	B4 (FPE)	B5 (DPE)
NON (n = 115)	0.31	0.15	1.40***	− 0.01	0.18	0.18
IND (n = 147)	0.85***	0.69***	0.74***	− 0.15	− 0.15	0.15
REC (n = 102)	2.48***	1.00***	1.31***	− 0.11	0.16	0.39*
RAT (n = 112)	2.01***	0.89***	0.21	0.08	0.10	0.19
All (n = 476)	1.31***	0.60***	0.91***	− 0.01	0.11	0.21**

*NON* non-cooperative group, *IND* indifferent group, *REC* reciprocal group, *RAT* rational group. *p < 0.05, **p < 0.01, ***p < 0.001.

## Discussion

Using unsupervised classification algorithms, we identified individual differences in the trajectory of social decisions and emotional responses to monetary rewards. Affective representation incorporating prosocial dimensions enabled the identification of individual differences and improved the explanatory power of GLMM models in predicting participants’ decisions. Specifically, prediction errors of dominance, valence, and reward dimensions positively predicted the recipients’ decision to accept. Subgroup analyses confirmed the dominance dimension’s utility, as individuals who showed different emotional responses to identical monetary rewards exhibited distinct economic responses to emotional experiences.

Participant decisions and emotional experiences were paired with the proposer’s offers within the same trial. Consequently, a comprehensive linkage was established between individual social decision-making, the contextual reciprocity to which the individual belongs, and an individual’s emotional response to the reciprocal atmosphere.

Previous studies reported a positive relationship between perceived reciprocity about a partner and the decision to maintain favourable interaction^1,3,39,40^. The subjective sense of reciprocity, which was embodied as a reward PE, has been extensively studied for its contribution to associative learning^4,5,41–43^. Furthermore, anticipatory emotions^6–8^ and expectation violations^10,12^ influence human decision-making within the social context.

To our knowledge, the generalization of partial relationships to establish a comprehensive link that spans the entirety of emotion–decision–reciprocity remains unexplored. DARM facilitates the investigation of momentary emotions during social decision-making processes. While Heffner, Son, and FeldmanHall attempted to establish the average trajectory of emotion after making specific decisions, their analyses did not consider emotion variability according to different levels of perceived reciprocity.

By clustering individuals based on the trajectory of economic decisions and emotional experiences in the face of variable offer levels, we showed that social context sharing equivalent reciprocity can result in divergent economic decisions and emotional experiences. Whether our clustering result based on shared reciprocal context can be generalised to context-independent cases remains debatable. Consequently, future studies should be conducted that modify the task so that the proposer’s offers are not identical across participants.

In our study, the valence, dominance, and reward PEs influenced social decisions, whereas the arousal and focus PEs did not. This observation aligned with the findings presented in Heffner, Son, and FeldmanHall^10^, where the valence PE had a more pronounced effect than the reward and arousal PEs.

The arousal PE had a nonsignificant influence on participant choices. Literatures on interoception and decision-making consistently indicates that physiological arousal positively predicts non-social decision-making^21,44,45^. During social interaction, higher interoceptive accuracy during heartbeat counting tasks strengthened coupling between anticipatory physiological arousal and UG decision making^46^, stabilized UG decision making through improved emotion regulation^47^. Heightened interoceptive awareness resulting from mindful meditation led to increased UG acceptance behaviours^48^ and moral behaviours during the temptation to lie card game^49^.

We interpreted our findings to support the claim that the impact of emotion PEs was contingent on the context^10^. In the previous study, participants at risk of depression exhibited a weakened relationship between the emotion PE and punitive behaviours. As our sample participants’ average depression (PHQ-9) scores corresponded to a moderately severe level, the nonsignificant contribution of arousal PE was consistent with previous reports.

Furthermore, we exemplified the usefulness of representing emotions using additional affective dimensions of social nature in classifying individuals and predicting their monetary decisions. These dimensions were adopted based on social psychology literature. As a two-dimensional affect grid that only incorporates interoceptive dimensions of valence and arousal has been widely used, multidimensional affective representation capturing social variance has been rarely explored. Adopting reward PE and all four affective dimensions significantly improved the explanatory power of GLMM models.

We also identified individuals who showed distinct trajectories of emotional experiences and differential weights of emotions on economic decisions. The non-cooperative participants were more likely to reject offers when their level of pleasure did not meet their expectations. Given a consistently low valence and other affective dimensions, this group may be in a state of negative, deactivated emotion, preventing them from adjusting interactions with partners until experiencing a relief of current displeasure. Moreover, reward PE had a nonsignificant contribution to responder choices. Hence, to change this group’s UG behaviour, interventions should focus more on how pleased they feel rather than optimizing the reward.

The rational group increased punishment only for unexpectedly low offers. Thus, this group’s behaviour was influenced solely by economic rationality without being swayed by their emotion about their partners. Notably, this group also exhibited consistently high level of emotions and the highest average scores in state anxiety, emotion regulation, and shame-proneness (Supplementary Table S8). A possible explanation is that they more actively regulated their emotions and were more attuned to discrepancies between their self-image and moral standards. Considering previous reports that meditation and improved emotion regulation predicts increased UG acceptance behaviours^47,48^ and altruistic behaviours^49^, these cognitive efforts might be required to maintain their positive emotional state.

The indifferent group showed a more complex relationship. They rejected more often when they were either less pleased or less rewarded than expected. This relationship was also the weakest of all groups (Table 3). Considering relatively neutral values of emotions during the task, this group may have been in a state of emotional detachment from their partners.

The group with the most complex emotion trajectory (REC group) also exhibited the most complex relationship between emotion PEs and social decisions. While their affective trajectories followed the variation of the proposer’s offers, the GLMM results suggested they changed their actions considering their emotional state independently of the reward PE. It is believed that fairness sensitivity is a distinctive trait of human nature^50,51^ and has a bidirectional impact on predictive emotions^20^. We think that the reciprocal group’s GLMM results gives support in such thinking.

Appropriate feature selection is important to tackle the heterogeneity problem of mental states by identifying subset populations^25^. Psychiatric classification models, including the Diagnostic and Statistical Manual of Mental Disorders 5 (DSM-5), are established based on case–control studies. They assume homogeneity among disease populations, which is not supported by empirical evidence^52–55^.

Notably, different emotional clusters identified in this study did not differ in their level of psychopathology ratings, except for rational groups, which also exhibited an equivalent level of depression and generalised anxiety to other groups (Supplementary Table S8). A comparison of acceptance behaviours and credits earned between participants with different levels of psychopathology scores also failed to reach significance (Supplementary Table S9). Our results support the claim that psychiatric diagnosis models are underspecified^25^. These variables can significantly vary independently of individuals’ severity of psychopathology. Hence, we suggest the need for a more context-specific model of human behaviours and emotional experiences. Affective representation adopting prosocial dimensions is a potential candidate.

The dominance dimension also appears promising for transdiagnostic phenotyping of individuals, as only the reciprocal group reacted to the dominance PE and changed their behaviours. Using the unsupervised classification algorithm implies that these are inherent subsets of participants. Participants were provided with clear definitions of affective dimensions and each emotion, thus eliminating the possibility of misinterpretation. We believe prosocial dimensions are necessary for the affective representation of emotions although their sufficiency remains to be explored.

Subgroup GLMM analyses for the relationship between emotion PEs and social decision-making suggest that each group reacted differently to monetary rewards and affective interventions. Thus, behavioural tasks can be individualised to influence participants’ social interactions in a disciplined manner based on their emotional characteristics. However, whether these group labels represent fluctuating affective states or more stable affective traits remains unclear. Repeated measurements are required for this inquiry^56^.

UMAP embedding results also showed a significant overlap between reward acceptance and emotion experience in the state space of reduced dimensions. Thus, the similarity between the temporal behaviours of social decisions and emotional experiences carried over to the manifold (Fig. 4). Although emotions were closely associated with rewards, they were not merely internal proxies for external reward experiences, thus supporting the conclusions presented in Heffner, Son, and FeldmanHall^10^.

Our study had several limitations. First, our task design used a conscious recollection of emotional experiences, excluding behavioural and physiological responses from the analyses. Notably, all emotion groups experienced a narrow range of arousal centred around the zero point. The task should be modified to contain more physiologically arousing inputs. Second, the use of pseudorandomised reward trajectory and distinct relationships between reciprocity, emotion, and social decisions necessitates further investigations under a divergent reciprocity context. Third, verification of participants’ self-reported characteristics and level of engagement was challenging due to the use of online recruitment methods. We believe that the universality of emotional experiences and concerns regarding the heterogeneity problem in psychiatry partially offsets the limitations of using an online platform. Fourth, the study was not pre-registered due to the exploratory nature of our findings. Fifth, momentary emotion labels can be predicted from social decision trajectories to predict prosocial behaviours further. Relationships between each prosocial emotion category and participants’ social decisions are of significant interest to psychological science researchers and the general audience. Sixth, the study assumed a one-way influence from reciprocity to social decisions mediated via emotional experiences, excluding the behavioural influences on participants’ emotions. Finally, we could not replicate the reported influence that preceding trials’ decisions have on subsequent trials^15^.

## Conclusions

In this study, we established a comprehensive link between predictive emotions, social decision-making, and perceived reciprocity of a partner by adopting prosocial affective dimensions. We also proposed a novel, data-driven classification algorithm to identify individuals with distinct economic decision-making and emotional experiences. Affective dimensions capturing aspects of social interaction aided both the classification and prediction problems of human behaviour and emotion. Further studies adopting more physiologically arousing stimuli and individual-specific reciprocity are warranted.

### Supplementary Information

Supplementary Information.

## Data Availability

All data and analysis scripts supporting the findings of this study are currently available at https://github.com/NarlKim/edr_review. For requests pertaining to the study's data and analysis scripts, readers are kindly invited to contact jwk1921 at kaist.ac.kr.
