# Supplementary material for: Prosocial emotions predict individual differences in economic decision-making during ultimatum game with dynamic reciprocal contexts
Source: Sci Rep. 2024 May 18;14:11397. doi: 10.1038/s41598-024-62203-y (PMC11102497; doi:10.1038/s41598-024-62203-y)
Supplement: Supplementary file 1 — Supplementary Information. [file 41598_2024_62203_MOESM1_ESM.pdf]

Supplementary Information for:

Prosocial Emotions Predict Individual Differences in Economic Decision-Making during Ultimatum Game with Dynamic Reciprocal Contexts

Table of Contents

Supplementary Methods **3**

Participants 3

Emotion Classification Task 4

Dynamical Affective Representation Mapping with Ultimatum Game 7

Clinical Ratings Questionnaires 10

Unsupervised Clustering of Reward Acceptance and Experienced Emotions 11

Generalised Linear Mixed Model to Predict Social Decision Making 17

Power analysis for sample size calculation 18

Supplementary Results 20

K-means Clustering Algorithm to Detect Individual Differences of Social Decision-making based on Increasing Level of Offer Fairness 20

Autoencoder Neural Network Classifier Identification of Central Tendency of Emotions based on Proposer's Offer among Individuals 20

Emotion PEs with Posocial Dimensions and Reward PE Predict UG Decisions 23

Individuals Differing in their Economic Response to Predictive Emotions 25

Supplementary Discussion 25

Co-independence between Emotion Experience Clusters and Depression/anxiety 25

Usefulness of Mutidimensional Affective Representation in Representing Prosocial Emotions 26

Supplementary Discussion for Limitations 27

1. Supplementary Methods

1.1. Participants

1.1.1. Eligibility criteria

Exclusion criteria : Participants were excluded if 1) their age was not within the range of 18 to 65 years, 2) they did not provide or revoked consent, or 3) they did not complete the task. Of the 636 participants recruited, 89 returned before completing the emotion classification task and 51 did not complete the entire experiments, leaving 496 intact responses apart from some missing values. Further, 19 participants retracted their consent upon completing the task and one participant did not provide consent. The remaining 476 participants were considered for the subsequent analyses.

1.1.2. Demographic variables

During the preliminary prescreening stage, prior to starting the experiments, we collected demographic data from participants. The collected demographic information included age, gender, the highest level of education attained, ethnicity, marital or relationship status, current employment status, presence of any ongoing mental health conditions or mental illnesses, presence of any ongoing long-term health conditions or disabilities, and nationality. Participants provided this information by selecting from predefined categories provided by the Prolific platform. Nationality data was classified into five regions of Africa, America, Asia, Europe, and Oceania according to (Nations 2019). Participants were given the choice to withhold any piece of information by opting out of consent for specific items. A summary of the participants' demographic details can be found in the main text (Table 1).

1.2. Emotion classification task

At the beginning of experiment, the participants were asked to rate 18 different discrete emotions using valence, arousal, focus, and dominance to evaluate the utility of the affect dimensions in representing emotions (Fig. 1A). Nine basic emotions (Happy, Sad, Afraid, Disgusted, Angry, Surprised, Satisfied, Relaxed, Sleepy) and nine moral emotions in adjective form were considered to be of interest (Ashamed/shameful, Guilty, Embarrassed, Proud, Righteously angry, Contemptuous, Morally disgusted, Elevated, Grateful).

Eight of the basic emotions, except for fear, were drawn from the octant of emotion classification plots from (Heffner and FeldmanHall 2022) for enhanced separation, as they occupied distinct regions dividing dynamical ARM measurement into octant. Because five of the eight emotions overlapped with six basic emotions (Ekman, 1971) the authors included fear, resulting in nine basic emotions. Nine moral emotions were adopted from moral emotion study (Tangney, Stuewig et al. 2007) (Table S1).

Table S1. Classification of Nine Moral Emotions (Tangney, Stuewig et al. 2007).

Valence	Focus	
Positive	Self	Pride
	Others	Elevation, Gratitude
Negative	Self	Shame, Guilt, Embarrassment
	Others	Righteous anger, Contempt, Moral disgust

1.2.1. Task instructions

Instructions for Emotion Classification Task

In this experiment, you will be asked to rate emotions with four different categories: Valence, Arousal, Focus, Dominance.

Valence is measure of how pleasant or unpleasant an emotion is. Positive sign indicates state of being pleasant, while negative sign indicates state of being unpleasant. The magnitude, or absolute value indicates the degree of being pleasant or unpleasant. Zero value means the state of being neither pleasant nor unpleasant.

Arousal is measure of how energising or soporific an emotion is. It represents the degree of psychologically and physiologically being aroused, rather than intensity of emotion. Positive sign indicates state of being aroused, while negative sign indicates state of being calm or drowsy. The absolute value or magnitude indicates the degree of being aroused or drowsy. Zero value means neither exciting/arousing nor calming/settling.

Focus is how much the emotion feels directed to oneself or others. It represents which of the two, oneself or others, an emotion is relevant to. Positive sign indicates state of being towards oneself, while negative sign indicates state of being towards others. The absolute value or magnitude indicates the degree of being towards oneself or others. It can range from 'completely towards others' (-4) to 'completely towards oneself' (+4). Zero value means the emotion has no particular direction or is equally relevant to both sides.

Dominance is the degree of control one has over the emotion. It represents the degree of being dominant over or submissive to the emotion of interest. Positive sign indicates state of being dominant over emotion, while negative sign indicates state of being submissive to emotion. The absolute value or magnitude indicates the degree of being in/out of control. It can range from 'completely out of control' (-4) to 'completely in control' (+4). Zero value means neither completely out of control, nor completely in control.

You will rate valence and arousal for each of the emotions by clicking on the two dimensional affect grid. All of the emotion words will be presented in adjective forms.

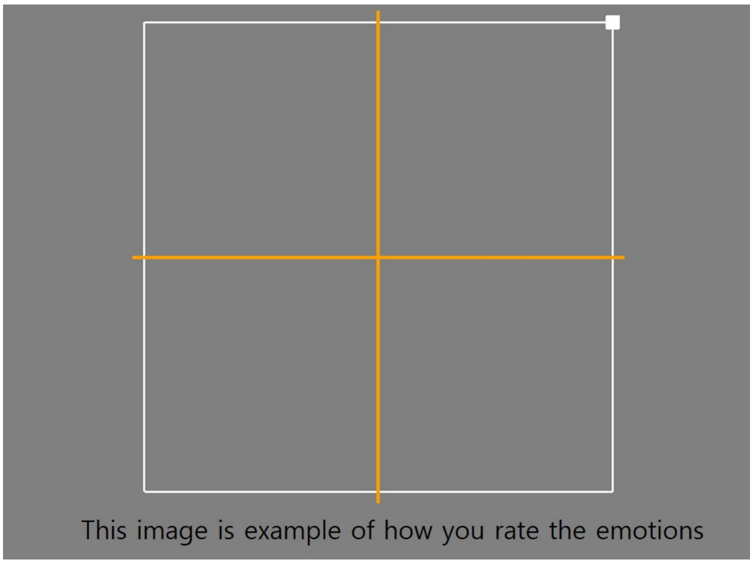

You will rate focus and dominance for each of the emotions separately. You should rate them by clicking on the respective scales. All of the emotion words will be presented in adjective forms.

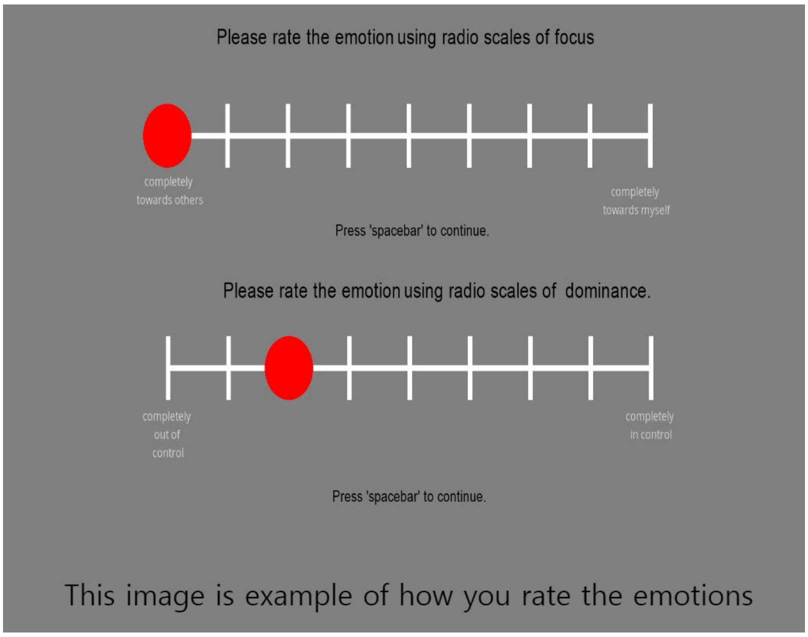

1.3. Dynamical affective representation mapping with ultimatum game

1.3.1. Task instructions

Instructions for Dynamical ARM with Ultimatum Game

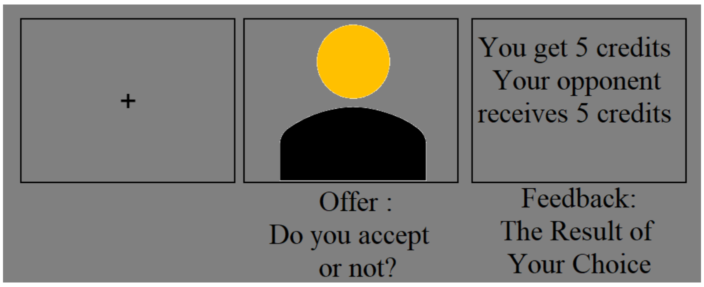

In this part of the experiment, you will make economic decisions regarding offers from other players. However, you will not come into contact with your fellow players outside of your brief economic interaction. In each round, you will receive offer from another player. You will never interact with the same fellow players.

The players will split $10 each round, and make an offer in $0-9 range. You will receive credits that you will exchange for dollars at the end. The exchange rate is: 1 credit = 1 cent. The corresponding amounts resulting from upcoming games with other participants in the study will be paid out to you after the study is completed. You must enter the completion code at the end to get the credits earned.

Your task is to make decision each round to accept or reject the offer that is split by another player. If the offer is accepted, both players, you and the proposer, will be credited with the corresponding amount. If the offer is rejected, no one will be credited with any amount.

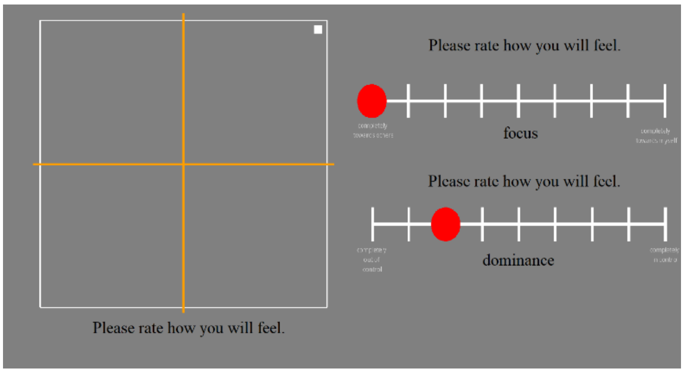

At the beginning of each round, you will be asked to respond : 1) How much money you EXPECT to be offered. 2) How you WOULD feel with the expected offer. Your expected emotion will be rated using dimensions of valence, arousal, focus, dominance, as explained earlier.

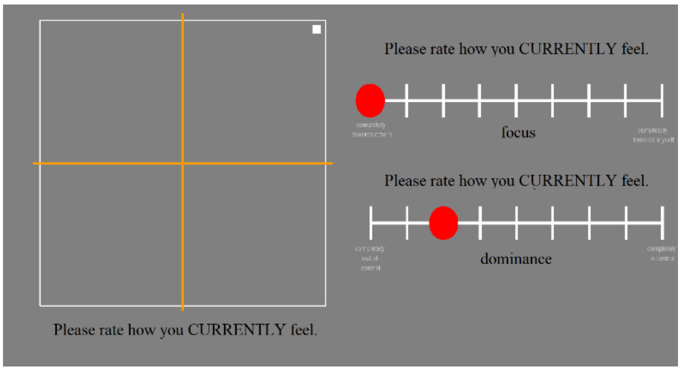

After you give response of your expected offer and emotion, an offer will be made from another player. Then you will be asked to respond how you feel AT THAT MOMENT using dimensions of valence, arousal, focus, and dominance. After you give response of your current emotion, you will be asked to decide either to accept the offer, or reject the offer. These processes will be repeated multiple times.

Figure represents the group to which another player belongs. To accept a corresponding offer, press ‘2’ key while the offer is displayed. To reject a corresponding offer, press ‘4’ key while the offer is shown. You have a maximum of three seconds for each decision.

If you understand the procedures, please continue to practice rounds.

Example Trials

The offer is “selfoffer”.

1) If participants failed to respond within three seconds : Please decide faster! Since you have not decided, the offer has been rejected.

2) If participants accepted offer within three seconds: You will get "selfoffer" credits. Your opponent gets "10 - selfoffer" credits.

3) If participants rejected offer within three seconds: You will get 0 credit. Your opponent gets 0 credit.

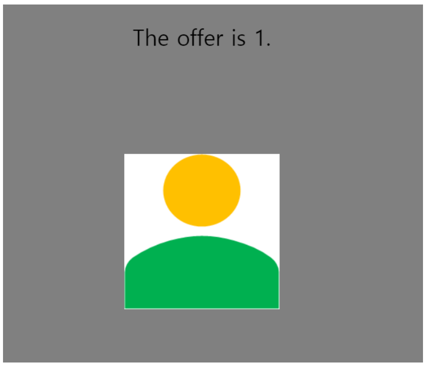

After six example trials : These were the practice rounds. Do you have any more questions? Otherwise, please start with the task now.

1.4. Clinical ratings questionnaires

Participants emotional states were investigated self-report questionnaires. Variables of interest were level of depression (Patient Health Questionnaire 9, PHQ-9), generalised anxiety (Generalised Anxiety Disorder 7, GAD-7), state anxiety (State-Trait Anxiety Inventory, STAI-X-1), emotion regulation (Emotion Regulation Questionnaire, ERQ), and proneness to shame and guilt (Personal Feelings Questionnaire 2, PFQ-2). Missing values in each rating were imputed with the average of each participant's intact responses (Table S2).

Table S2. Summary of Participants' Self-Reported Clinical Rating Scores

Ratings	Mean	S.D.	Min	25%	50%	75%	Max
PHQ-9	17.0	5.9	9.0	12.0	16.0	21.0	36.0
GAD-7	13.4	5.1	7.0	9.0	12.0	16.0	28.0
STAI-X1	42.6	5.2	27.0	39.0	42.0	46.0	66.3
ERQ							
Total	46.6	8.2	15.0	41.0	47.0	52.0	68.0
Control	29.9	6.7	6.0	27.0	30.0	34.0	42.0
Express	16.7	5.5	4.0	12.0	17.0	21.0	28.0
PFQ-2							
Total	33.4	12.6	16.0	23.5	31.0	41.0	73.0
Shame	20.8	7.7	10.0	15.0	20.0	26.0	45.0
Guilt	12.6	5.5	6.0	8.0	11.0	16.0	30.0

1.5.Unsupervised clustering of reward acceptance and experienced emotions

1.5.1. Selecting optimal number of clusters for reward groups

Optimal number of clusters for expected reward and reward acceptance trajectories were identified via fviz_nbclust() function in R's factoextra package. Arguments were desiganted as FUNcluster = 'kmeans', method = 'wss'. From the within sum of score plotting results, k = 4 was chosen to be optimal (Fig. S1–2)

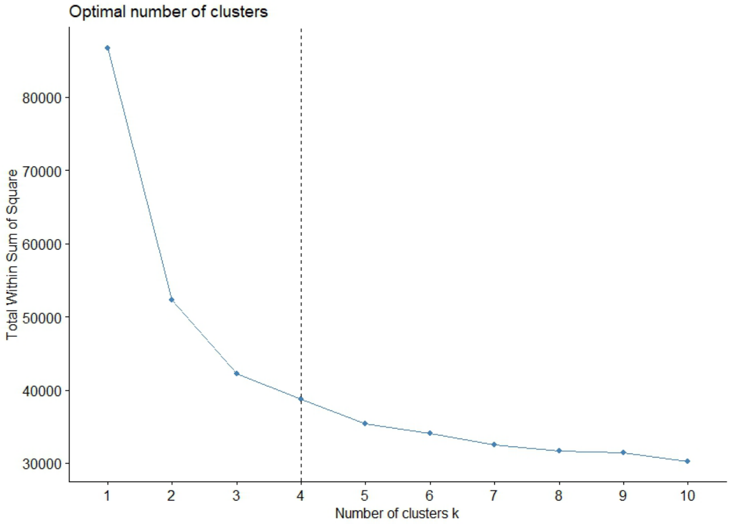

Fig. S1. The within sum of squares values for k-means clustering of expected reward. The X-axis represents k values, while the Y-axis shows the within sum of square values for each corresponding k. k = 4 was automatically chosen according to genu method.

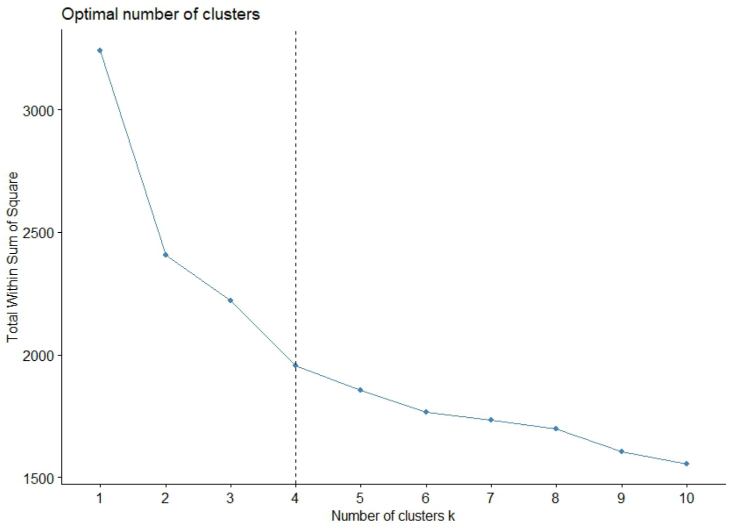

Fig. S2. The within sum of squares values for k-means clustering of reward acceptance. The X-axis represents k values, while the Y-axis shows the within sum of square values for each corresponding k. k = 4 was automatically chosen according to genu method.

1.5.2. Selecting optimal number of clusters for emotion groups

Since expected emotions and experienced emotions were multi-dimensional time series with temporal fluctuations, we applied t-distributed stochastic neighbor embedding (t-SNE) on the bottleneck of autoencoder to explore the inherent temporal patterns of emotion using TensorFlow (Fig. S3). The inertia plot revealed an elbow point at k = 4, and the silhouette score plot showed local maxima at k = 4 for both expected emotions and experienced emotions, respectively (Fig. S4).

The autoencoder for expected emotions consisted of a 3-layer encoder and a 3-layer decoder. The input shape for the encoder was '30 * 4'. The second layer of the encoder had '200' neurons, with activation set to 'selu', bias_initialiser to initialisers.TruncatedNormal(mean = 0, stddev = 1.0), and kernel_regulariser to regularisers.l2(0). The third layer of the encoder had '200' neurons with 'selu' activation. The decoder accepted the third layer output of the encoder as its input. Its second layer's configuration included '200' neurons, 'selu' activation, an input shape

of [200], bias_initialiser set to initialisers.TruncatedNormal(mean = 0, stddev = 1.0), and regularisers.l2(0) for kernel regularization. The decoder's output layer was a dense layer with an output shape of '30 * 4', reshaped to [30, 4]. The optimiser used was 'adam' with parameters: learning_rate = 0.001, beta_1 = 0.9, beta_2 = 0.999, and epsilon = 1e-07. Learning was conducted for 1000 epochs with early stopping set at 789 epochs.

The autoencoder for experienced emotions also consisted of a 3-layer encoder and a 3-layer decoder. The input shape for the encoder was '30 * 4'. The second layer of the encoder had '200' neurons, with activation set to 'selu', bias_initialiser to initialisers.TruncatedNormal(mean = 0, stddev = 0.5), and kernel_regulariser to regularisers.l2(0). The third layer of the encoder had '150' neurons with 'selu' activation. The decoder accepted the third layer output of the encoder as its input. Its second layer's configuration included '200' neurons, 'selu' activation, an input shape of [150], bias_initialiser set to initialisers.TruncatedNormal(mean = 0, stddev = 0.5), and regularisers.l2(0) for kernel regularization. The decoder's output layer was a dense layer with an output shape of '30 * 4', reshaped to [30, 4]. The optimiser used was 'adam' with parameters: learning_rate = 0.001, beta_1 = 0.9, beta_2 = 0.99, and epsilon = 1e-07. Learning was conducted for 1000 epochs with early stopping set at 563 epochs.

The original multi-dimensional time series data was standardised to fall within the range of [0, 1] using scikit-learn's MinMaxScaler. The mean squared error (MSE) between the raw time series and the output of the autoencoder was 0.0039 for expected emotions and 0.00023 for experienced emotions.

We used dimensionality reduction for two primary reasons: first, to map the emotion trajectories within a state space that encompasses all participant responses over time; and second, to enhance the visualization of these emotion trajectories. We transformed the participants' response data from a three-dimensional array with (samples, time steps, and

dimensions) into a two-dimensional array by combining the time steps and samples (samples * time steps, dimensions). This restructured data was then fitted into various dimensionality reduction algorithms. To identify the central point of each affect dimension, we applied the k-means clustering algorithm with a single cluster (k = 1), and these centroids were further processed using uniform manifold approximation and projection (UMAP). The resulting central points are indicated by 'x' markers in Fig. 4A.

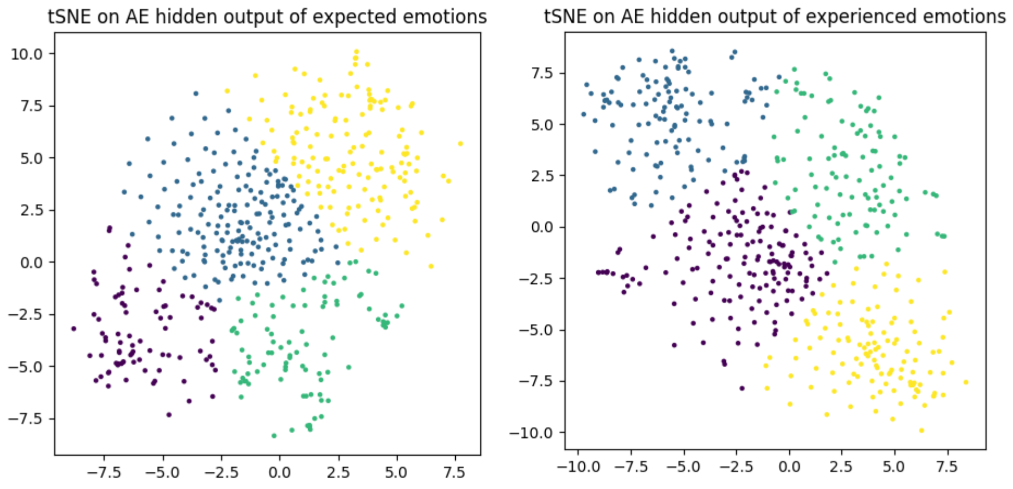

Fig. S3. The output from the bottleneck of the autoencoder's hidden layer for expected emotions (left) and experienced emotions (right) trajectories is depicted. Each individual dot represents the time series of a specific participant. Groups of dots with the same color in both diagrams indicate semantically matched trajectories; however, these groups do not necessarily consist of the same participants.

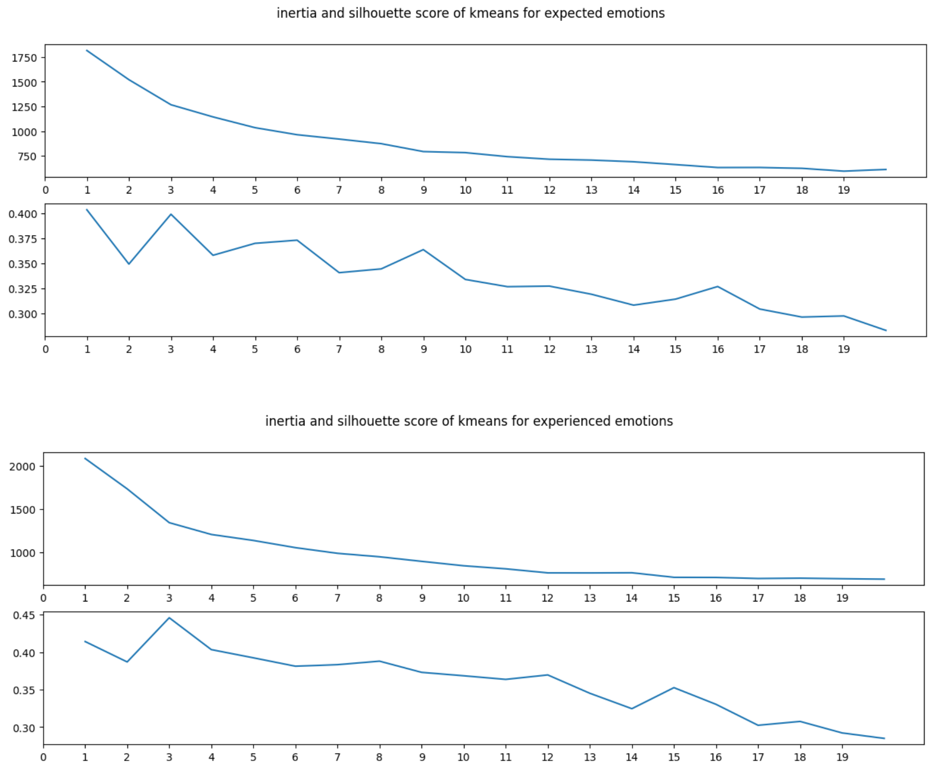

Fig. S4. The inertia and silhouette scores for clustering of (top) expected emotions and (bottom) experienced emotions based on tSNE embedding of autoencoder bottleneck layer, using different k values, are presented. The X-axis represents (k-1) values, while the Y-axis shows the inertia score (upper plot) and silhouette score (lower plot) for each corresponding k.

We employed various dimensionality reduction techniques such as PCA, t-SNE, MDS, and tPHATE, as described in Fig. S6–7. We chose UMAP over PCA, tSNE, and MDS due to its superior ability to delineate clusters. While tPHATE provided as clear demarcations among groups as UMAP, it did not result in clearer delineation of cluster spatial representations. Conversely, UMAP adeptly illustrated not only the distinct spatial patterns for static trajectories but also the varied and broader spatial patterns for reciprocal groups. Computational burden of tPHATE was also much greater than that of UMAP.

The average value of expected emotions and experienced emotions for each group was compared using one-way ANOVA and post-hoc TukeyHSD. Except for experienced dominance dimension comparison between IND and REC group (mean difference 0.12, p = 0.0639), affect dimensions were significantly different in every possible combination of groups. Estimates of affect dimension by emotion groups are summarised in Table S3.

Table S3. Mean Values of Affect Dimensions for Each Expected Emotion and Experienced Emotion Group.

	Valence	Arousal	Focus	Dominance
Expected emotions				
PES (N = 82)	-78.8	-19.9	-1.89	-2.08
FAIR (N = 166)	25.8	4.5	0.37	0.29
RECe (N = 92)	38.4	27.8	0.10	-1.72
OPT (N = 136)	124.3	37.1	2.04	1.74
Experienced emotions				
NON (N = 115)	-142.3	-17.7	-2.43	-2.51
IND (N = 147)	-40.7	-11.0	-0.05	-0.87*
RECx (N = 102)	-23.9	21.8	-0.62	-0.99*
RAT (N = 112)	83.5	14.4	1.73	1.41

Note : *Pairs for which the Tukey HSD test did not reach significance at $p < 0.05$ are marked with asterisks. Abbreviations : PES, pessimistic group, FAIR, fair-expectation group, RECe, reciprocal group in expected emotions, OPT, optimistic group, NON, non-cooperative group; IND, indifferent group; RECx, reciprocal group in experienced emotions; RAT, rational group.*

1.6. Generalised linear mixed model to predict social decision making

A generalised linear mixed model (GLMM) was applied on the participants' decision for each trial as a dependent variable. Reward PE, valence PE, arousal PE , focus PE, and dominance PE were used as predictors. Participants were treated as the grouping variables. The models were nested based on the magnitude of Akiake Information Criteria (AIC) value reduction and compared using AIC for predictive accuracy (see Equations 1–5, Table S4). The significance of model comparisons was tested via likelihood ratio tests. Intra-individual-level correlations between the predictors of the model, and their variance inflation factor (VIF) for collinearity were investigated (Table S6–7)

M1a : Decision ~ Valence PE … Eq.1

M2a : Decision ~ Valence PE + Reward PE … Eq.2

M3a : Decision ~ Valence PE + Reward PE + Dominance PE … Eq.3

M4a : Decision ~ Valence PE + Reward PE + Dominance PE + Focus PE … Eq.4

M5a : Decision ~ Valence PE + Reward PE + Dominance PE + Focus PE + Arousal PE … Eq.5

For each experienced emotion group, we repeated procedures of 1) building most complex model, 2) setting simplest model according to beta coefficient, 3) nesting models according to magnitude of AIC value reduction, 4) model comparison via AIC and likelihood ratio test, 5) repeated measure intra-individual correlation and VIF calculation for reliability of GLMM relationship.

To investigate the predictive ability of the winning GLMM M5a models across groups, we generated simulated data representing the probability of accepting reward, predicted from actual values of reward PE and emotion PEs. This process corresponds to parameter recovery

with only one iteration. The results showed good predictive ability, with all models' AUC values exceeding 0.91. If we rounded off the predicted values, the models' accuracies were all greater than 0.82. See Fig. S8 for details.

Table S4. Comparison of Nested GLMM Models Predicting Participants' Acceptance Behaviour during UG based on Reward and Emotion Prediction Errors.

Model name	AIC	BIC	Loglik	χ^2	*df*	P
M1a	15041	15078	-7515.4			
M2a	14553	14621	-7267.4	495.83	4	<0.001
M3a	14212	14318	-7092.2	350.57	5	<0.001
M4a	14093	14244	-7026.3	131.75	6	<0.001
**M5a**	**14017**	**14222**	**-6981.6**	**89.37**	**7**	**<0.001**

Note : *Likelihood ratio test statistics were calculated to compare each model with its preceding best-performing model. This analysis encompassed 14,280 observations from 476 participants. The most successful model is highlighted in bold. The 'Bobyqa' optimiser was utilised for this analysis.*

1.7.Power analysis for sample size calculation

Statistical power analyses were conducted using the powerSim function from the simr package in R to determine the appropriate sample size. Heffner, Son and FeldmanHall (2021) found that reward prediction error, valence prediction error, and prediction error significantly influenced participant choices in their first experiment. This experiment involved 364 participants, and the significance was determined through a Z-test on the beta coefficients obtained from a generalized linear mixed model (GLMM).

We conducted power analyses by simulating participant selections using the GLMM model and observed values of valence PE, arousal PE, and reward PE. Assuming that the GLMM model accurately represents the actual data-generating process, the power value, which is the complement of the type II error, can be calculated by determining the proportion of simulations that result in statistical significance. We performed separate simulations for each predictor as a fixed effect (valence PE, arousal PE, and reward PE). Statistical significance for each simulation was determined using a Z-test. Additionally, we simulated participant selections with all predictors of the GLMM model as fixed effects. Statistical significance for each simulation was determined using a likelihood ratio test with the GLMM model having no fixed effects as the reduced model. The power value was calculated for sample sizes of 364, 50, and 100. Each simulation was repeated 100 times. The detailed results are presented in Table S10.

2. Supplementary Results

2.1. K-means clustering algorithm to detect individual differences of social decision-making based on increasing level of offer fairness.

We compared the mean acceptance proportion of given reward (p_{accept}) between each pair of reward acceptance groups. One-way ANOVA indicated significant differences in p_{accept} among reward acceptance groups ($F = 36.37$, $p < 0.001$). Post-hoc TukeyHSD confirmed significant differences between all group pairs except for IND – REC comparison (Table S5).

Table S5. Post-hoc TukeyHSD Results Comparing Average Acceptance Proportions between Reward Acceptance Groups

Groups	Difference	Lower bound	Upper bound	P adjusted
NON – IND	0.39	0.23	0.56	<0.001
NON – RAT	0.64	0.48	0.81	<0.001
NON – REC	0.30	0.14	0.46	<0.001
IND – RAT	-0.25	-0.41	-0.08	<0.001
IND – REC	0.10	-0.07	0.26	0.425
RAT – REC	0.34	0.18	0.51	<0.001

2.2. Autoencoder neural network classifier identification of central tendency of emotions based on proposer's offer among individuals.

Embedding the expected reward trajectory along the reduced state space of expected emotion indicated alignment with and dispersion from spatial representation of emotion experience groups. The stationary reward expectation groups (PES, FAIR, OPT in Fig. S5) occupied

regions similar to that of the matched expected emotion groups (the groups with same name in Fig. S5), whereas the dynamic groups (RECe in Fig. S5) exhibited similar patterns of more dispersed spatial representations that spanned the entire range of the UMAP emotion expectation space.

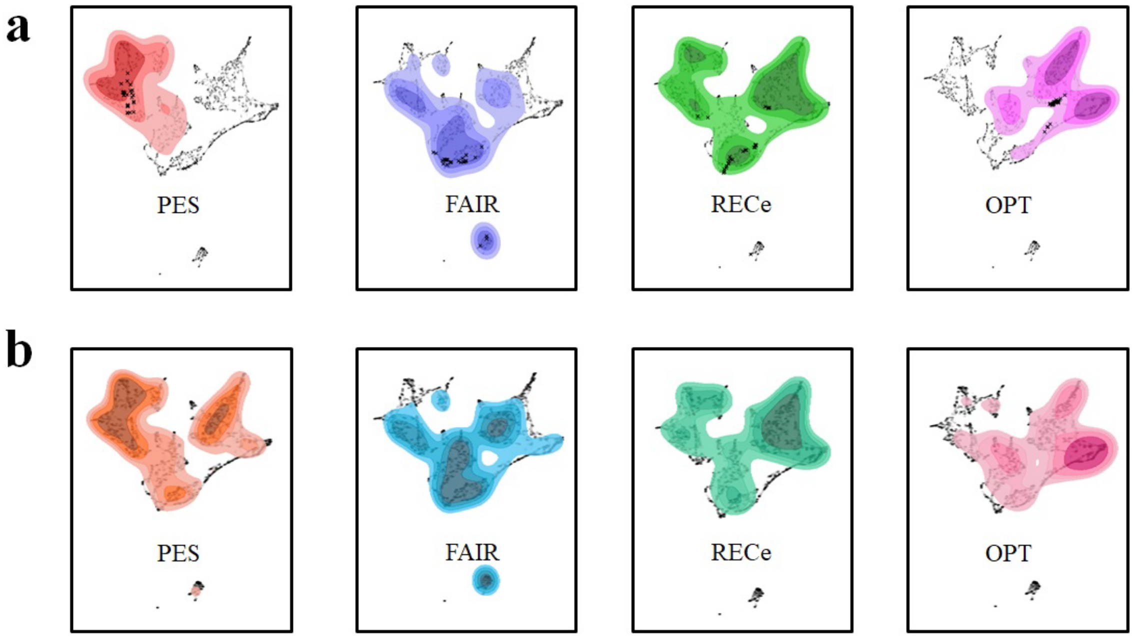

Fig. S5. Kernel density estimation (KDE) plot for (a) emotion expectation group and (b) reward expectation groups (bottom) on the UMAP (Uniform Manifold Approximation and Projection) embedding space of emotion. Each expected reward group occupied a region similar to that of the corresponding emotion group. Abbreviations : PES, pessimistic group; FAIR, fair group; RECe, reciprocal expectation group; OPT, optimistic expectation group.

We also applied multiple dimensionality reduction algorithms (PCA, t-SNE, MDS, UMAP, tPHATE) to the time series of expected and experienced emotions using scikit-learn, TensorFlow, Keras, umap, and tphate packages (Fig. S6–7). The kernel density estimation plot was visualised separately for each emotion group using Python's matplotlib and seaborn packages.

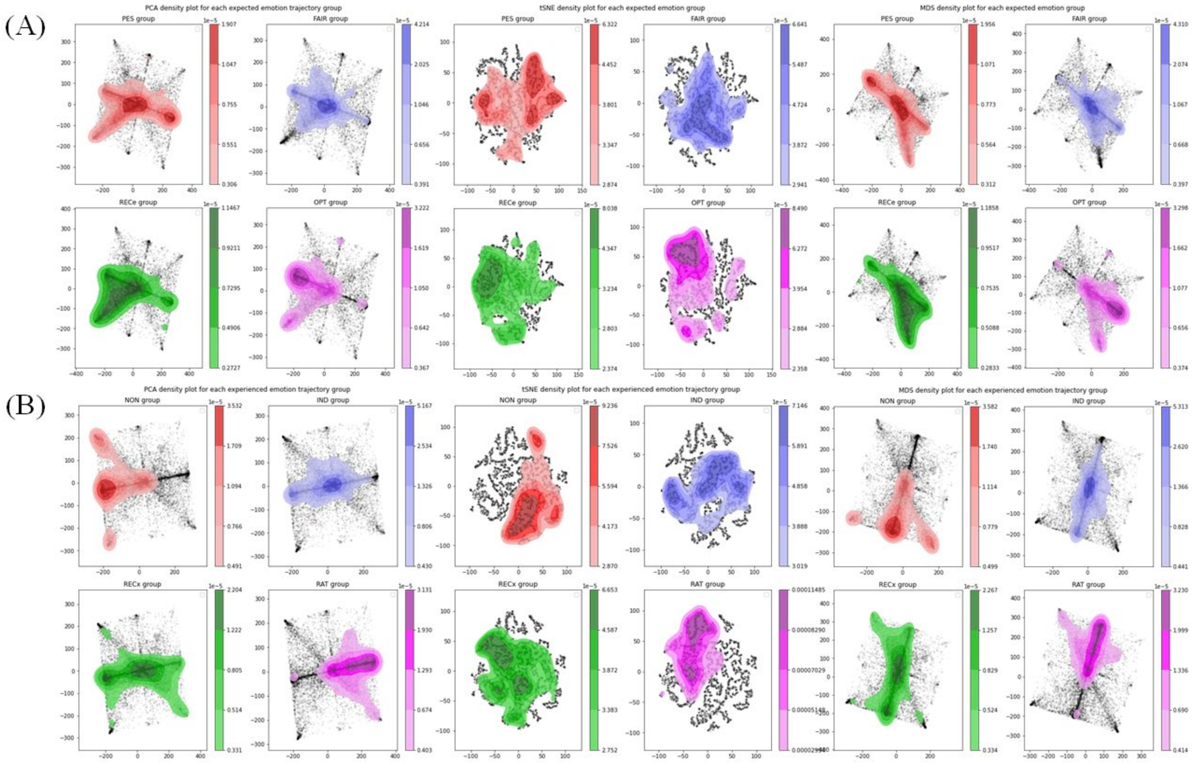

Fig. S6. **(left) PCA, (center) tSNE, (right) MDS embedding of (up) expected emotions and (down) experienced emotions.**

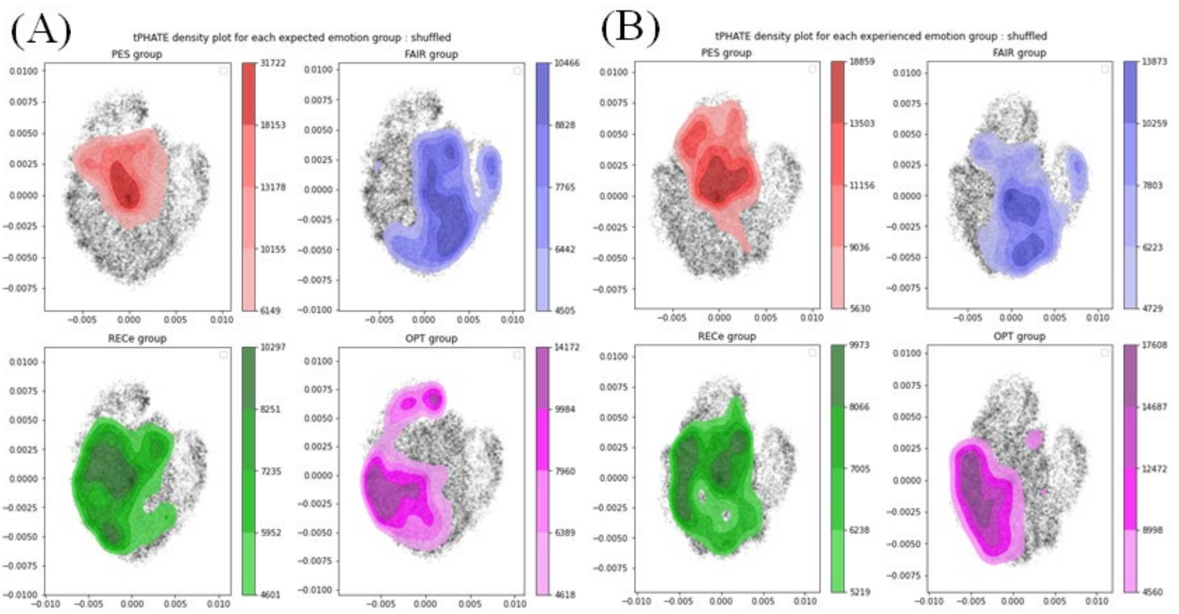

Fig. S7. Temporal PHATE provided as clear demarcations among groups as UMAP, but did not result in clearer delineation of cluster spatial representations.

2.3.Emotion PEs with prosocial dimensions and reward PE predict UG decisions

Predictors of the GLMM model m5a did not show high intra-individual-level correlations (Table S6), and their variance inflation factor (VIF) statistics indicated low collinearity with each other (Table S7). These results support the reliability of the relationship between the reward PE, emotion PEs, and the participants' social decisions.

Table S6. Repeated Measure Correlation of Predictor Variables at Intra-individual level.

	RPE	VPE	APE	FPE	DPE
RPE	1				N = 14,280
VPE	R = -0.06<br>P < 0.001	1			DF=13,803
APE	R =0.008<br>P < 0.001	R=0.14<br>P < 0.001	1		
FPE	R =-0.16<br>P < 0.001	r=0.43<br>P < 0.001	R=0.12<br>P < 0.001	1	
DPE	R = -0.38<br>P < 0.001	R=0.41<br>P < 0.001	R=0.11<br>P < 0.001	R=0.52<br>P < 0.001	1

Note : *R's rmcorr library was used for calculation.*

Table S7. Variance Inflation Factor of Predictor Variables in Model M5a

Predictor	VIF value
Reward PE	1.04
Valence PE	1.12
Arousal PE	1.08
Focus PE	1.21
Dominance PE	1.13

2.4. Individuals differing in their economic response to predictive emotions.

The predictive ability of the winning models was generally good, with all AUC values exceeding 0.9 (Fig. S8).

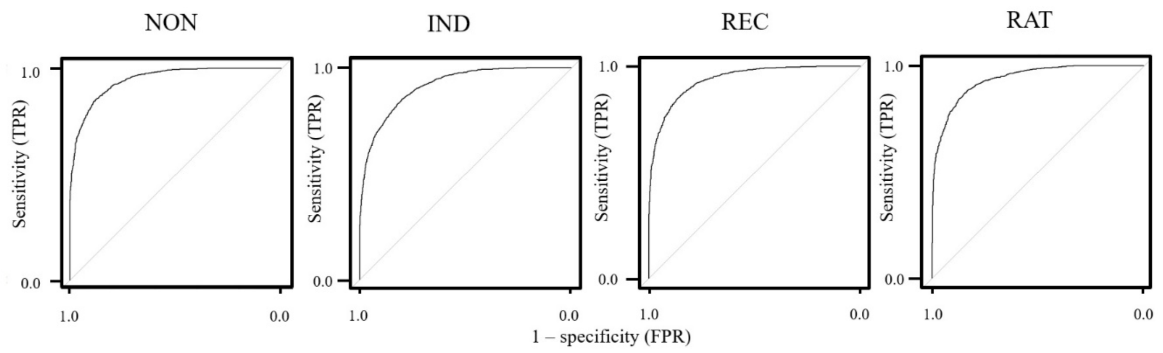

Fig. S8. Individualised GLMM models show good predictive ability for participants' social decision-making. AUC values were 0.938 (ENT), 0.945 (NON), 0.919 (IND), 0.940 (REC), 0.938 (RAT). When the predicted values of each model were rounded off, their accuracies were in the range of [0.829, 0.868] ; 0.857 (ENT), 0.868 (NON), 0.829 (IND), 0.871 (REC), and 0.866 (RAT). Abbreviations : ENT, entire participants; NON, non-cooperative group; IND, indifferent group; REC, reciprocal group; RAT, rational group.

3. Supplementary Discussion

3.1. Co-independence between emotion experience clusters and depression/anxiety

The psychopathology severities were compared between emotion experience groups. Only the rational group exhibited significant difference in state anxiety, ERQ total scores, ERQ expression subscores, PFQ-2 shame subscores from other groups (Table S8).

We also evaluated the effect of clinical ratings on social decision-making. First, we divided the participants based on the lower and upper quartiles of each rating. Subsequently, independent

t-tests were applied to compare paccept along all levels of offers for two groups. None of the 40 comparisons were significant (Table S9). Further, the earned credits were compared, and only the ERQ score groups showed trend-level differences ($p = 0.084$).

3.2.Usefulness of mutidimensional affective representation in representing prosocial emotions

To further evaluate the utility of focus and dominance in differentiating prosocial emotion labels, estimated probability density functions were plotted for the participants' ratings of nine prosocial emotions from ECT. We found supporting evidence for positive valence and self-focus for pride, and negative valence and other focus for righteous anger, contempt, and moral disgust. However, no evidence was found supporting self-focus for shame, guilt, embarrassment, nor was other focus supported for elevation and gratitude (Fig. S9). Specifically, representations of shame, guilt, and embarrassment were nearly identical.

Our results suggest that definitions for some moral emotions may need revisiting. Shame is commonly understood as a negative evaluation of the global self, guilt as a negative evaluation of specific actions, and embarrassment as a state of mortification arising from social predicaments. These definitions support the cognitive emotion theory's premise that emotions are functions of internal or external events, their situational meaning, and an agent's appraisal. Prosocial emotions, being complex affective states, seem to be significantly influenced by cognitive processes and extend beyond mere interoceptive or autobiographical dimensions.

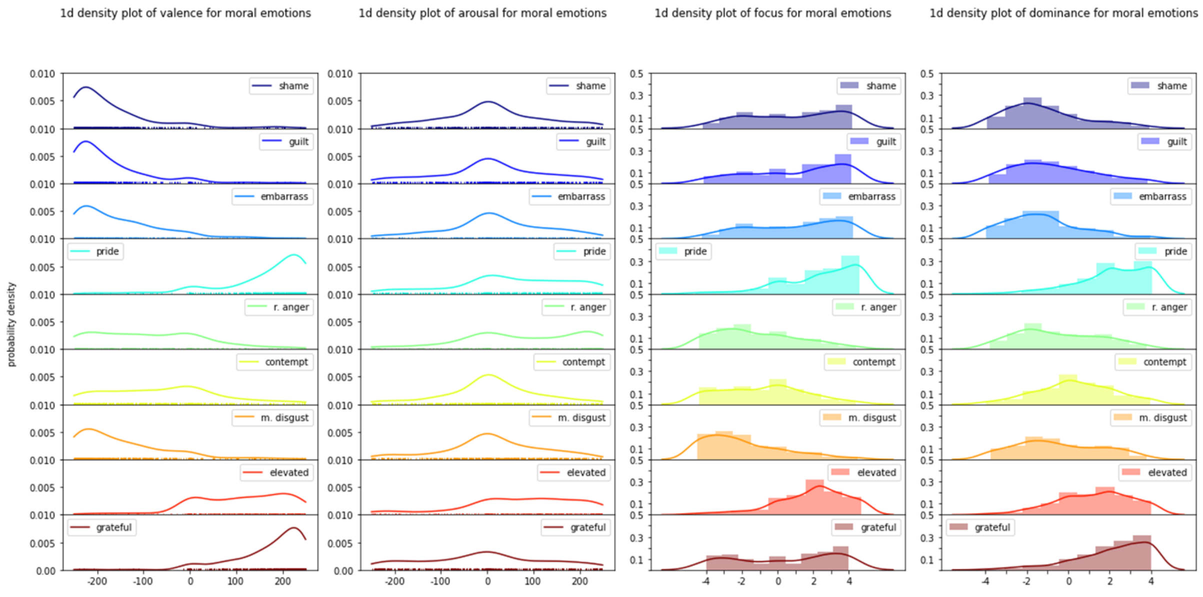

Fig. S9. Kernel density estimation plot of affect dimensions for nine moral emotions. Seaborn's distplot function was used with the following arguments : (hist = False, kde = True, rug = True, bins = 501, norm_hist = True) for valence and arousal, (hist = True, kde = True, rug = False, bins = 9) for focus and dominance.

3.3. Supplementary discussion for limitations

The Prolific platform collected demographic information prior to study participation. When participants initiated the experiment, the PsychoPy task opened in a new window, unlinked to their Prolific ID. Consent was only obtained upon completing the task, resulting in a mismatch between the number of participants who shared demographic information and those who consented to participate in the study.

Second, a relatively high severity of psychopathology ratings was noticeable. In fact, approximately 20% of the participants indicated that they had at least one on-going mental illness or mental health issue. The universality of emotion experiences did not preclude

populations with adverse mental health conditions, although stratified samples would be more desirable.

Table S8. Mean Values of Clinical Ratings for Each Experienced Emotion Group.

Experienced emotions	PHQ9	GAD7	STAI-X1***	ERQ*	ERQ Cont	ERQ exp†	PFQ2†	PFQ2 Shame*	PFQ2 Guilt
NON (N = 115)	17.7	13.8	41.8**	46.2	29.2	17.0	32.6	20.0*	12.5
IND (N = 147)	16.6	12.9	41.8 ***	46.6	29.9	16.7	32.8	20.6	12.3
REC (N = 102)	16.2	13.0	42.7	45.1*	29.5	15.6*	32.4	20.2†	12.2
RAT (N = 112)	17.4	13.9	44.2 **, ***	48.5*	30.9	17.6*	36.1	22.7†,*	13.4

Note : *Abbreviations: NON, non-cooperative group; IND, indifferent group; REC, reciprocal experience group; RAT, rational group.* †, $p < 0.10$; *, $p < 0.05$; **, $p < 0.01$; ***, $p < 0.001$

Table S9. Independent t-Test Results for Acceptance Proportion and Accumulated Credits Between Upper and Lower Quartile Groups of Clinical Ratings.

Psychopathology	Offer level								Earned credit
	0	1	2	3	4	5	6	7	
PHQ9	T=.521	T=.031	T=.386	T=1.494	T=1.028	T=.056	T=-.515	T=1.008	T=.806
	P=.603	P=.975	P=.700	P=.137	P=.305	P=.955	P=.607	P=.315	P=.421
GAD7	T=1.062	T=.474	T=.280	T=1.240	T=1.429	T=.661	T=-.152	T=.861	T=.836
	P=.289	P=.636	P=.780	P=.216	P=.154	P=.509	P=.879	P=.390	P=.404
STAI-X1	T=-1.909	T=-1.400	T=-.464	T=.247	T=.306	T=-1.298	T=-1.481	T=-1.144	T=.648
	P=.057	P=.163	P=.643	P=.805	P=.760	P=.196	P=.140	P=.254	P=.517
ERQ	T=-.242	T=-1.234	T=-.776	T=-.660	T=.848	T=.531	T=.199	T=.582	T=1.733
	P=.809	P=.219	P=.438	P=.510	P = .397	P=.596	P=.842	P=.561	P=.084
PFQ2	T=-.410	T=-.145	T=-.127	T=.488	T=-.317	T=-.707	T=-1.603	T=-.190	T=1.460
	P=.682	P=.885	P=.899	P=.626	P=.751	P=.480	P=.110	P=.849	P=.146

Note : *No entry survived FDR (False Discovery Rate) correction.*

Table S10. Power Analysis Results for Sample Size Calculation

	Valence PE	Arousal PE	Reward PE	Entire model
N = 50	82.0 [73.1–89.0]	92.0 [84.8–96.5]	91.0 [83.6–95.8]	100 [*96.4–100.0*]
	-1.5	1.0	-1.3	
N = 100	99.0 [94.6–99.9]	89 [81.2–94.4]	100 [96.4–100.0]	100 [96.4–100.0]
	-1.5	0.8	-1.5	
N = 364	100 [69.2–100.0]	100 [96.4–100.0]	100 [96.4–100.0]	100 [96.4–100.0]
(Entire data set)	-1.9	0.5	-1.1	

Note : *R's simr library was used for power value calculation. Each simulation was performed for 100 iterations. Power value in percentage, with 95% confidence intervals, as well as effect size for each predictor is given.*
